# Life expectancy and survival analysis for companion dogs and cats in Seoul, South Korea

**DOI:** 10.3389/fvets.2025.1532422

**Published:** 2025-04-30

**Authors:** Isaac Yang, Dae-Sung Yoo, Kyung-Duk Min

**Affiliations:** ^1^College of Veterinary Medicine, Chungbuk National University, Cheongju, Republic of Korea; ^2^College of Veterinary Medicine, Chonnam National University, Gwangju, Republic of Korea

**Keywords:** life expectancy, life tables, survival analysis, companion dogs, companion cats

## Abstract

**Background:**

Investigating life expectancy and mortality is crucial for the development of evidence-based health strategies for companion animals. However, relevant studies are lacking in South Korea, possibly because of challenges in collecting mortality data. In this regard, preliminary analyses were conducted to obtain life tables for companion animals in South Korea.

**Methods:**

The electronic records of six veterinary hospitals in Seoul, South Korea were examined. The data collected included breed, sex, spay/neuter status, date of birth, and date of death for all dogs and cats with a verifiable date of death since November 1, 2004 until December 31, 2022. After data preprocessing, descriptive statistical analysis was performed to summarize the demographics, and life tables and survival curves were created for dogs and cats. Cox proportional hazards regression was used to analyze the effects of demographic factors on survival.

**Results:**

The mean age of dogs at death was 3427.49 days. Spayed or neutered dogs had a significantly higher life expectancy than intact dogs. Mixed-breed dogs had a higher life expectancy than purebred dogs. For cats, the mean age at death was 1965.49 days, with spayed or neutered cats living significantly longer than intact cats. Purebred cats had a higher median survival than Mixed-breed cats. Spaying or neutering and breed significantly affected survival probabilities in both species.

**Conclusion:**

Our study provides insights into the longevity of companion animals in South Korea, and reveals that neutering and breed significantly influence life expectancy.

## 1 Introduction

Investigating the life expectancy and mortality at different ages is important in the development of evidence-based strategies to improve the health of companion animals (1). Life tables tabulate the probability of death and life expectancy at various ages in a given population. Therefore, it is necessary to create and properly utilize life tables. For example, the optimal age for regular medical checkups can be proposed based on reliable life tables that suggest ages with higher risk of mortality. In addition, life tables can help identify factors that cause health problems in population groups with similar characteristics, such as breed groups, and take appropriate action to mitigate the problem (2) or can support the design of a sustainable public or private insurance system that can mitigate the owners’ financial burden. However, the number of relevant existing studies is insufficient.

One of the major reasons for the lack of such studies could be the limitations of the collection of data on mortality. In contrast to humans, there is a lack of well-established mandatory registration systems for the births and deaths of companion animals in the veterinary field. A practical method for acquiring reliable data is the use of veterinary medical data from small animal clinics. Many medical records are required, and effective data collection systems that facilitate the merging of records from multiple veterinary clinics are available; these include the Small Animal Veterinary Surveillance Network (3) and VetCompass (4). A previous study investigating life tables in the United Kingdom used this system (5). Additionally, well-established insurance systems can be used to investigate life tables like the one conducted in Japan (6).

However, in South Korea, it is even more challenging to study the life tables of companion animals for several reasons. First, data from companion animal registration systems are unreliable because of their low levels of completeness. Although the animal registration system for companion dogs has been mandatory since 2014 (7), this system does not manage the registration related to the death of companion dogs, and the number of registered companion dogs and cats is significantly lower than the national census (8), implying that it is challenging to understand the birth and death of companion animals using registration data. Second, merging data from multiple veterinary institutions is difficult because effective data collection systems have not been implemented in South Korea. Third, the data quality of mortality records could be unreliable because the motivation for veterinary professionals to record death events is lacking. In contrast to the requirements for human doctors, reporting of the mortality of companion animals is not the legal responsibility of veterinarians in South Korea.

Despite previous studies in other countries, specific studies in South Korea are required owing to prospective heterogeneity between countries. Because influential factors for life expectancy, such as dominant breeds (9), prevalent diseases, and natural and social environments, differ (10), similar patterns of life expectancy are unlikely between countries. Therefore, we conducted an analysis to develop life tables for companion animals in South Korea. As a preliminary analysis, we selected six veterinary hospitals that voluntarily participated in this study.

## 2 Materials and methods

The electronic medical record (EMR) databases of six veterinary hospitals in Seoul, South Korea were used for this study. The data collected included all dogs and cats with a verifiable date of death between November 21, 2004 and December 31, 2022. Consequently, the records of 2,024 dogs and 1,213 cats were collected.

The characteristics used in the study were the de-identified animal identifier, breed, sex, spay/neuter status, date of birth, date of death, and age at death (the difference between the date of death and date of birth in days). The characteristics utilized in the study were managed separately from free text entries of the medical record within the EMR database, thus the study data cannot be used to identify the animals or the individuals associated with veterinarians or animal owners.

If the string comprising a dog’s breed record included ‘mix’ or ‘mik-su’ (Korean for ‘mix’), the dog was considered a mixed breed; all other dogs were considered pure breeds. If a cat’s breed was domestic short hair or the string comprising the record contained ‘mix’ or ‘mik-su,’ the cat was considered a mixed breed; all other cats were considered pure breeds.

Descriptive statistical analyses were performed to summarize the demographic characteristics of the study population. In order to ascertain the life expectancy at each age interval and to present a visual representation of the manner in which deaths occurred across the age interval, life tables and survival curves were created for the study population. To create the life table, we used the method described in the study by Teng et al. (5). Supplementary Table 2 lists the notations, definitions, and equations for each parameter in the life table.

Thereafter, we created separate survival curves for each subgroup of the study population and performed log-rank tests. Subgroup-specific life tables were created for the subgroups that showed statistically significant differences in the log-rank test. In addition, considering that infertility surgery is usually performed between 4 and 6 months after birth, a separate study population was created with a mortality age of more than 365 days, and the survival time was analyzed and a log-rank test was performed. Finally, Cox proportional hazards regression analysis was performed to analyze the effect of dog and cat demographics on survival.

Microsoft SQL Server 2008 and SQL Server Management Studio (version 19.3, Microsoft Corporations, Redmond, WA, USA) were used for data extraction and preprocessing from the databases of each veterinary hospital. Analysis and visualization of the study population were performed using Rstudio (version 2023.12.1 + 402) and the R program (version 4.3.2, R Foundation for Statistical Computing, Indianapolis, IN, USA). In R, the packages Tidyverse (version 2.0.0) and Ggplot2 (version 3.4.4) were used for descriptive analysis and visualization, and the packages Survival (version 3.5–7) and Survminer (version 0.4.9) were used for life table and survival curve construction and Cox proportional hazards model construction.

## 3 Results

The mean age of the dogs at death in the study population was 3427.49 days with a standard deviation (SD) of 2187.82. For cats, the mean age at death was 1965.49 days (SD = 1890.18). When broken down by spay/neuter status, 979 dogs (48.37%) in the study population were intact or neutered. In terms of sex, 895 (44.22%) were male and in terms of breed, 1823 (90.07%) were purebred. In the cat study population, 444 (36.60%) were intact or neutered. A total of 665 cats (54.82%) were male. A total of 575 (47.40%) were pure breeds. The demographic characteristics of the study population are summarized in Table 1.

**Table 1:** Demographic characteristics of the study populations.

Characteristic	Value	
	Canine	Feline
Age in days at death, mean (SD)	3427.49 (2187.82)	1965.49 (1890.18)
Sex, Spay/Neuter Status, n (%)		
Male, Intact	295 (14.58)	227 (18.71)
Male, Neutered	600 (29.64)	438 (36.11)
Female, Intact	684 (33.79)	217 (17.89)
Female, Spayed	445 (21.99)	331 (27.29)
Breeds (Pure-breed or Mix-breed), n (%)		
Pure-breed	1823 (90.07)	575 (47.40)
Mix-breed	201 (9.93)	638 (52.60)

The life table for the entire study population is summarized in Supplementary Tables 3, 4. In the life tables created for the dog and cat study populations, the life expectancy at age 0 was 9.390 years in dogs and 5.384 in cats. The probability of a dog dying between the ages of 0 and 1 year was 0.161. After age 1 until age 10, the probability of dying at each age was <0.1, so the dog’s life expectancy was higher at age 1 (10.123 years) than at age 0 (9.390 years). When the dog reached the 17–18-year age range, the probability of death exceeded 0.5, and the life expectancy at that point was 1.117 years. For cats, the probability of death between the ages of 0 and 1 year was 0.302. From age 1–10, the probability of death at each age was <0.15. Therefore, as in dogs, cats have a higher life expectancy at age 1 (6.544 years) than at age 0 (5.384 years). When cats reached the 17 to 18-year age range, the probability of death exceeded 0.5, and the life expectancy at that point was 1.290 years.

**Figure 1:**
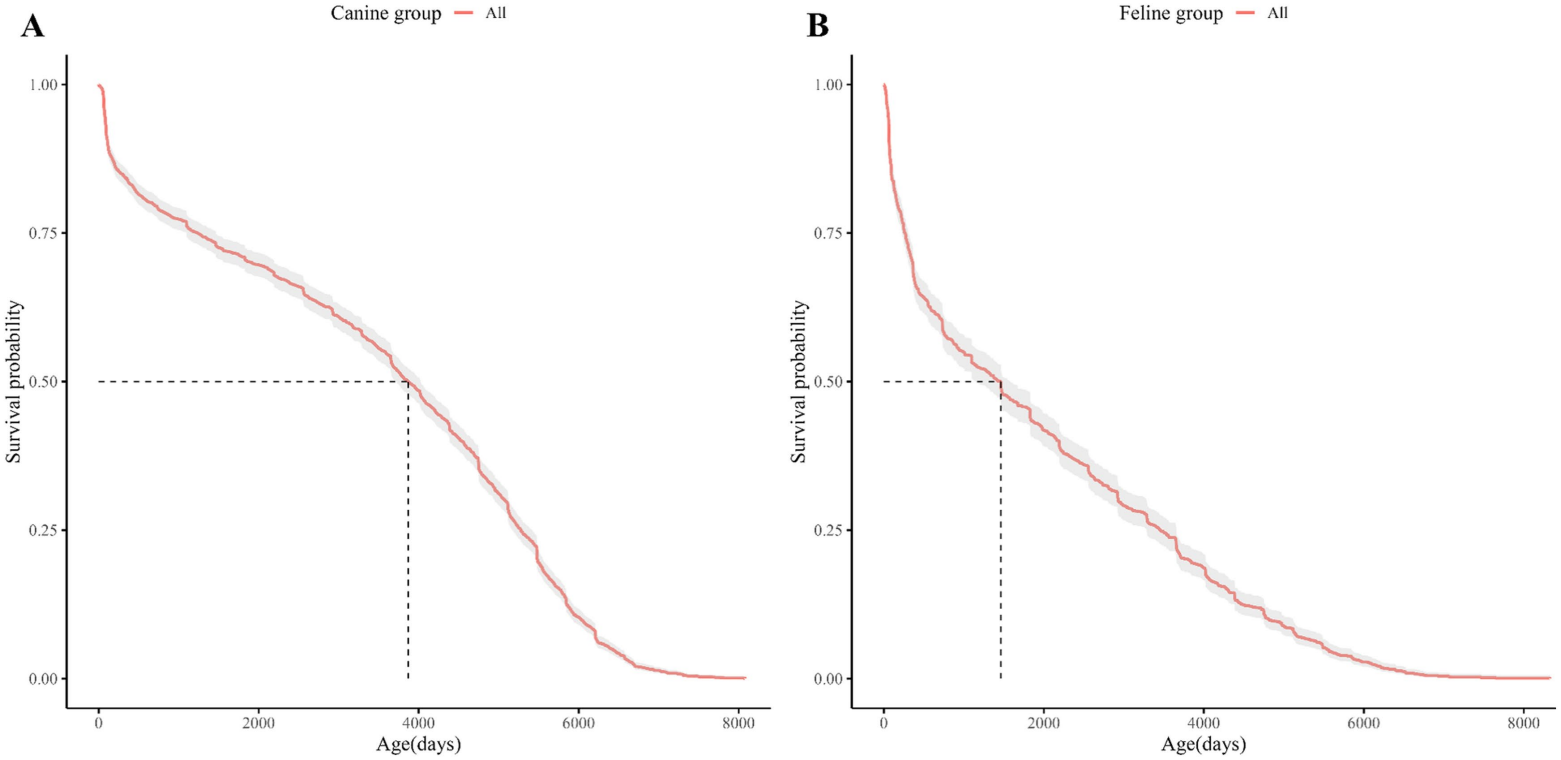
Kaplan Meier curve of the entire study population of dogs and cats. **(A)** The Kaplan–Meier curves for dogs, **(B)** for cats. The horizontal axis shows the age of the dogs or cats and the vertical axis shows the cumulative survival probability by age of the dogs or cats. The red graph shows the Kaplan Meier curve for dogs and the blue graph for cats. The shading around the graphs represents the 95% confidence intervals. The vertical and horizontal dashed lines represent median survival.

**Figure 2:**
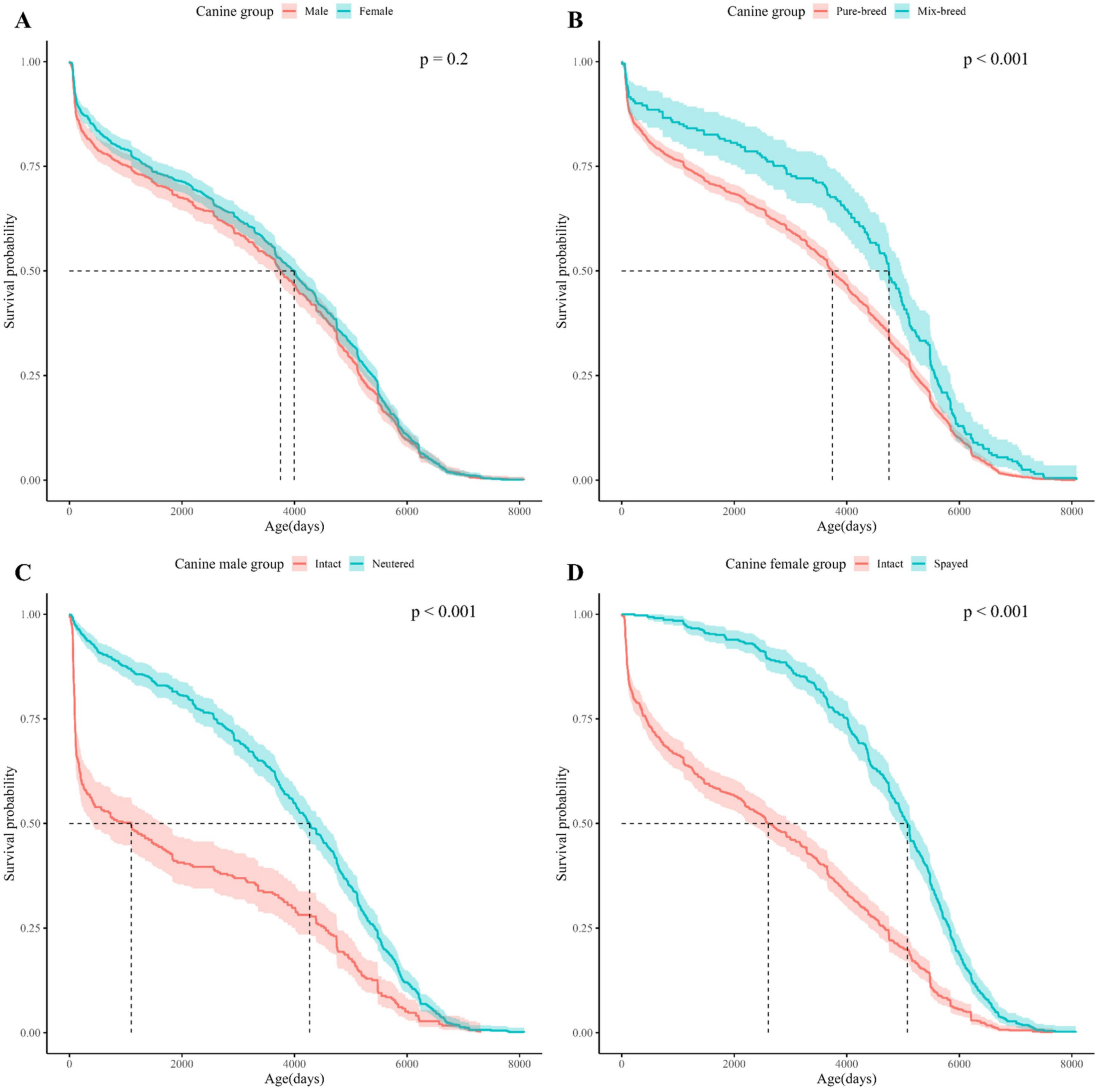
Kaplan Meier curves for dogs survival based on sex, breed, and spay/neuter status. The horizontal axis shows the age of the dogs in the subgroup and the vertical axis shows the cumulative survival probability by age of the dogs. **(A)** Shows the Kaplan–Meier curves for dogs by sex, **(B)** for dogs by breed, **(C)** for male dogs by neuter status, **(D)** for female dogs by spay status. In the upper right corner of each subfigure, the *p*-value of the Log-rank test performed on each subgroup is shown.

**Figure 3:**
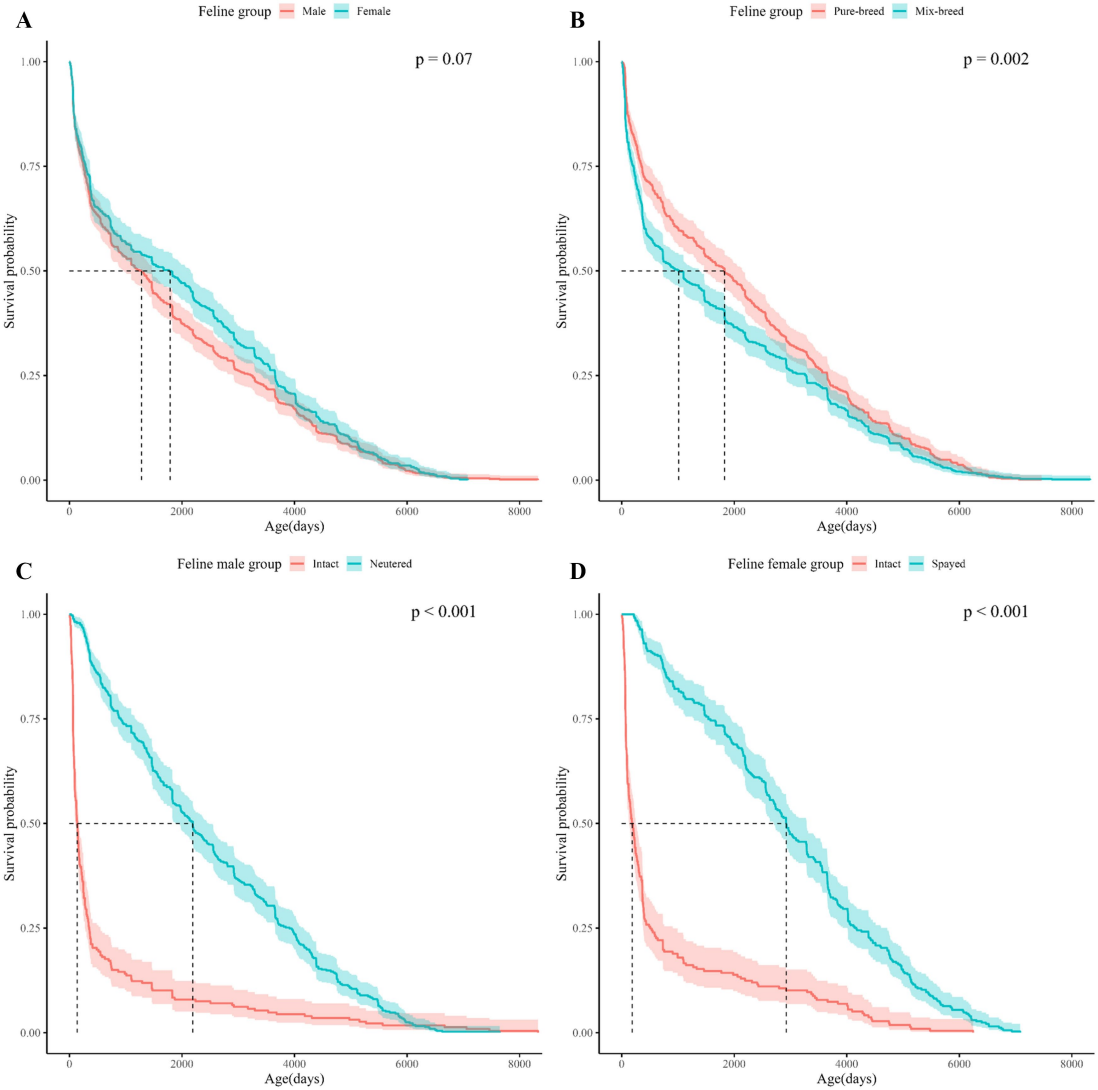
Kaplan Meier curves for cats survival based on sex, breed, and spay/neuter status. The horizontal axis shows the age of the cats in the subgroup and the vertical axis shows the cumulative survival probability by age of the cats. **(A)** Shows the Kaplan–Meier curves for cats by sex, **(B)** for cats by breed, **(C)** for male cats by neuter status, **(D)** for female cats by spay status. In the upper right corner of each subfigure, the *p*-value of the Log-rank test performed on each subgroup is shown.

Kaplan–Meier (KM) curves for the dog and cat study populations are shown in Figure 1. The age at which the survival probability became 0.5 was 3,869 days in dogs and 1,460 days in cats (median survival age). The KM Curve for the study population divided by demographic characteristics (spay/neuter status, sex, and breed) is shown in Figures 2, 3.

Log-rank tests between demographic characteristics (spay/neuter status, sex, and breed) showed significant differences in survival between subgroups for spay/neuter status and breed (Table 2). Considering that infertility surgery is usually performed between 4 and 6 months after birth, a separate study population was formed with a mortality age of more than 365 days, and the survival period was analyzed and a log-rank test was performed (Supplementary Table 1). Life tables of dogs and cats by subgroup based on spay/neuter status, sex, and breed are presented in Supplementary Tables 5–16.

**Table 2:** Kaplan–Meier survival analysis (Log-Rank Test) for the demographic characteristics of the study populations.

			Overall survival times (days)		
	Variables	Cases	Mean	Median	*p*-value
Canine	Sex				0.2
	Male	895	3,320	3,750	
	Female	1,129	3,512	3,996	
	Neuter Status (in Males)				<0.001
	Intact	295	2,149	1,097	
	Neutered	600	3,896	4267.5	
	Spay Status (in Females)				<0.001
	Intact	684	2,700	2,606	
	Spayed	445	4,759	5,079	
	Breeds				<0.001
	Pure-breed	1,823	3,351	3,743	
	Mix-breed	201	4,119	4,748	
Feline	Sex				0.07
	Male	665	1,852	1,280	
	Female	548	2,103	1,789	
	Neuter Status (in Males)				<0.001
	Intact	227	607	137	
	Neutered	438	2,496	2,192	
	Spay Status (in Females)				<0.001
	Intact	217	747	185	
	Spayed	331	2,991	2,925	
	Breeds				0.002
	Pure-breed	575	2,168	1,827	
	Mix-breed	638	1,782	1012.5	

In the comparison between subgroups, the subgroup with the largest difference in median survival age was neuter status in dogs, spay status in cats: for dogs, the difference in median survival age between intact and neutered male dogs was 3170.5 days. For cats, the mean survival age difference between intact female cats and spayed female cats was 2,740 days. Due to the extreme statistic of a median survival age of 137 days for unspayed cats and 185 days for unneutered cats (Figures 3C,D), both mean and median survival ages are presented in Table 2 to provide information on the distribution of data by subgroup.

**Figure 4:**
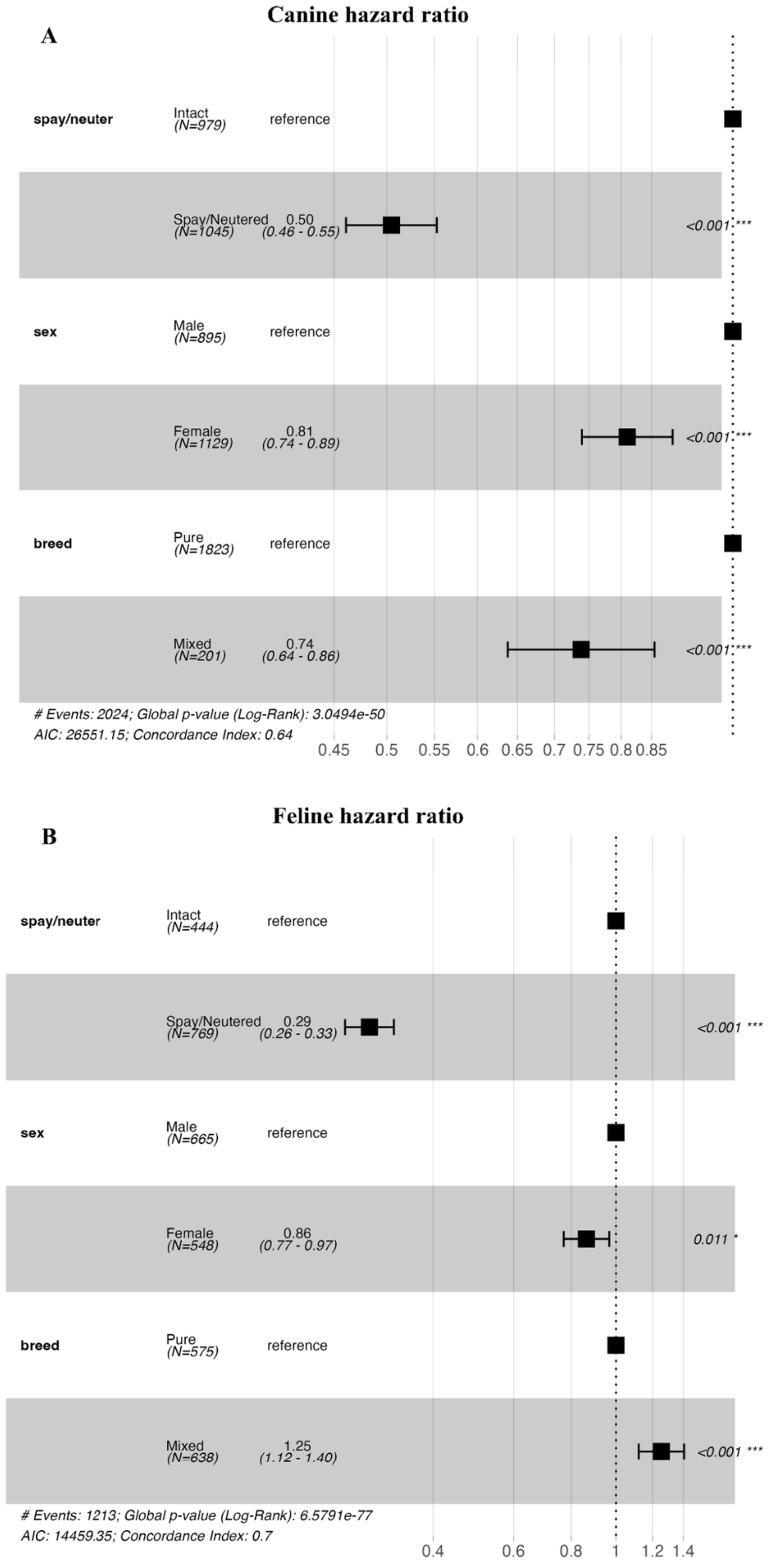
Cox regression analysis for dogs and cats based on spay/neuter status, sex, and breed. **(A)** Is a forest plot of the hazard ratio of dogs by spay/neuter status, sex, and breed. **(B)** Is a forest plot of the hazard ratio of cats by spay/neuter status, sex, and breed. In each subfigure, the horizontal axis shows the hazard ratio. The black squares represent point estimates of the hazard ratio for each subgroup, and the horizontal line across the black squares represents the 95% confidence interval.

The results of the Cox regression analysis using demographic characteristics of the dog and cat study populations, and the estimated regression coefficients and confidence intervals are shown in Figure 4. Cox regression analysis of the demographic characteristics of the study population showed that the hazard ratio for spayed or neutered versus intact or neutered dogs was 0.50. In a Cox regression analysis, the relative risk of an independent variable on the occurrence of an event is assessed by a hazard ratio. A hazard ratio of 0.5 means that the group exposed to the variable is half as likely to experience a death as the group not exposed to the variable, meaning that spayed or neutered dogs had a positive effect of reducing the chance of death by 50% compared to intact or neutered dogs. For male versus female dogs, the hazard ratio was 0.81. For mixed breed versus purebred dogs, the hazard ratio was 0.74 (Figure 4A, global *p*-value <0.001). For intact versus spayed or neutered cats, the hazard ratio was 0.29. For male versus female cats, the hazard ratio was 0.86. For mixed-breed versus purebred cats, the hazard ratio was 1.25 (Figure 4B, global *p* < 0.001).

In summary, the results of the Cox regression analysis indicate that, for both dogs and cats, spayed or neutered animals show a lower risk of mortality compared with intact animals, and female animals have a lower risk of mortality compared with male animals. However, in dogs, mixed-breed dogs demonstrate a lower mortality risk than purebred dogs, whereas in cats, mixed-breed cats show a higher mortality risk than purebred cats.

## 4 Discussion

Our study provides comprehensive insights into the longevity of companion animals in South Korea, highlighting significant differences between dogs and cats. For dogs, the mean age at death was 3427.49 days, with spayed or neutered dogs exhibiting a higher life expectancy than their intact counterparts. Mixed-breed dogs had a higher life expectancy than purebred dogs, which underscores the influence of genetic diversity on health outcomes. Similarly, for cats, the mean age at death was 1965.49 days, with spayed or neutered cats living significantly longer than intact cats. However, in contrast to the findings for dogs, the study revealed that mixed-breed cats had a lower median survival than purebred cats.

This study showed that the general longevity of companion dogs and cats in South Korea is lower than that in the USA and the UK. A descriptive epidemiological study using clinical records in the USA showed that the life expectancies of companion dogs and cats were 12.69 and 11.18 years, respectively (11). In contrast, studies in the UK revealed longevities of 11.23 and 11.74 years, respectively (5, 12). There may be systematic factors that cause a shorter life expectancy. For example, unlicensed medical practice by owners without veterinary consultation or prescriptions is more prevalent in South Korea (13). In addition, owners in these countries may have better pet insurance than those in South Korea (14, 15), and health insurance may increase their willingness to pay for veterinary care (16), which would subsequently increase the lifespan of companion animals. Further investigations are required to elaborate on this issue.

Our findings indicate that spayed or neutered animals have a longer lifespan than their intact counterparts. While the impact of spaying and neutering on the lifespan of dogs and cats is an ongoing debate, as it can vary by sex, breed, and the specific circumstances of each animal, our findings are generally consistent with existing consensus that spaying and neutering has the potential to reduce the risk of certain diseases and increase life expectancy (5, 11, 12). First, spaying and neutering eliminates or significantly reduces the risk of numerous reproductive diseases (17, 18). In males, neutering prevents testicular cancer and significantly lowers the risk of prostate disorders. In females, spaying eliminates the risk of ovarian and uterine cancers and drastically reduces the incidence of mammary tumors, which are often malignant in dogs. Spaying or neutering extends dogs’ lifespans by eliminating these potential health threats. Second, neutered dogs are generally less aggressive and less likely to roam (19). This behavioral change significantly reduces the risk of injuries from fights, accidents through roaming (such as being hit by cars), and other misadventures. Behavioral problems are a common cause of euthanasia in dogs; thus, reducing these issues can indirectly contribute to increased longevity. Although hormonal changes can also lead to certain health risks, such as a potential increase in the risk of obesity due to a reduced metabolic rate (20, 21), their overall impact on longevity remains positive. However, a more in-depth analysis of the study group, which was over 1 year (365 days) old at the time of death, revealed that neutering had no clear effect on lifespan compared to spaying in both dogs and cats. Consequently, this result should be interpreted with prudence and further research is necessary to investigate additional factors that may influence the lifespan of male companion animals.

A lower hazard ratio for females than for males was suggested in this study. This finding is consistent with a previous study that showed a longer lifespan in female than in male animals of various wild species (22). Several factors, ranging from biological to behavioral, could contribute to these differences. Some previous studies have suggested that females have inherent biological advantages that contribute to their longevity. This may include more robust immune responses that offer greater resistance to certain diseases and infections (23). In addition, a review revealed that heterogametic genders tend to have a higher risk of deleterious mutations in the sex chromosome (unguarded X hypothesis) (24). Behaviorally, male animals are often more prone to risk-taking than are female animals. Although this association is controversial in humans (25), it has been demonstrated in animal studies (26) to include greater aggression, higher propensity to roam, and more frequent involvement in fights with other animals. Such behaviors significantly elevate the risk of accidents, injuries, and infectious diseases, which can adversely affect lifespan. Females generally exhibit more cautious behaviors, reducing these risks (27).

Cox regression also revealed that purebred dogs face higher risks than mixed breed dogs, potentially reflecting the influence of genetic diversity on disease susceptibility and overall health. This finding supports the hypothesis that genetic factors play a crucial role in determining lifespan and suggests that breeding practices can significantly affect canine longevity. This result is consistent with previous studies (28) and can be attributed to several factors grounded in genetic and health considerations. This phenomenon is often discussed in the context of what is known as “hybrid vigor” or heterosis, which suggests that crossbreeding leads to an increase in genetic diversity, thereby enhancing the overall health and resilience of mixed-breed dogs. Mixed-breed dogs benefit from a broader genetic pool, which reduces their susceptibility to inherited diseases that are often prevalent in purebred populations owing to selective breeding practices. Pure breeds are typically selected for specific traits that can inadvertently concentrate deleterious alleles and increase the prevalence of heritable conditions such as hip dysplasia, heart disorders, and certain cancers (29). In contrast, our study found that purebred cats have a longer lifespan. Despite the different population and study design, this is different from previous findings in the UK (30). This could be explained by the fact that the overwhelming majority of stray cats in Korea are mixed breeds, and it is common for stray cats to be adopted and kept as in-house cats. Although there is a paucity of large-scale studies examining overall longevity and causes of death in cats, it is evident that stray cats are at an elevated risk of contracting infectious diseases such as feline leukemia virus (FeLV) in comparison to in-house cats. Furthermore, the presence of an FeLV infection can have a profound impact on the lifespan of a cat (31). A 2018 Animal Protection National Awareness Survey conducted by the South Korean government (Ministry of Agriculture, Food and Rural Affairs) revealed that 20.6% of cat-owning respondents had brought in stray cats and kept them as companion animals. In contrast, only 4.6% of dog owners indicated that they had adopted stray dogs and kept them as companion animals. The localized nature of South Korea, where it is common to bring in stray cats that are relatively vulnerable to infectious diseases and keep them as companion animals, may be a contributing factor to the shorter life expectancy of mixed-breed cats in this study. In fact, past studies have shown that mixed breed cats in Korea have higher mortality rates from external causes compared to purebred cats (32). In general, younger mixed-breed cats tend to roam outside more, increasing their risk from external sources compared to purebred cats. This characteristic of mixed-breed cats may shorten their lifespan.

Although this study offers significant insights, it has some limitations. First, the data were obtained from veterinary clinics and are of a retrospective nature, which may introduce inherent bias in the sample selection process. For instance, it is possible that unhealthy animals may have been overrepresented in the sample, and there may be issues with the accuracy of historical records. The low percentage (2.9%) of all records collected that were utilized in the analysis without being excluded, and the difference in the percentage between dogs and cats, may be indicative of these issues. Second, environmental factors and variations in veterinary care, which may significantly influence lifespan, were not controlled for in this study. However, because these were not confounding factors in our analysis, there were no systematic biases in the results. Third, data were only collected from six institutions in Seoul if the date of death of the animal was recorded in a structured format. It is often the case that information related to animal deaths is recorded in free text in electronic medical records. Therefore, this small, spatially biased sample may not represent the general companion animal population in South Korea. Fourth, the study did not collect data that could be or are significantly correlated with longevity, such as an animal’s weight or body size, vaccination status, whether the dog is a brachycephalic breed, or the cause of death (31, 33).

Nonetheless, our study underscores the importance of considering a wide range of biological and care-related factors when assessing the lifespan of companion animals. These findings highlight specific actionable interventions such as spaying/neutering and provide a basis for further research on genetic influences and breeding practices that could enhance the health and longevity of companion animals in South Korea. Future research should incorporate these variables to provide a more comprehensive picture of the factors affecting longevity. Prospective studies could provide more controlled and accurate data to further refine our understanding of how genetics, care, and environmental factors interact to determine the lifespan of companion animals.

## Data Availability

The data analyzed in this study is subject to the following licenses/restrictions: the data that support the findings of this study are available from the corresponding author, Min KD, upon reasonable request. Requests to access these datasets should be directed to Kyung-Duk Min, kdmin@cbnu.ac.kr.
