# Supplementary material for: Life expectancy and survival analysis for companion dogs and cats in Seoul, South Korea
Source: Front Vet Sci. 2025 Apr 30;12:1532422. doi: 10.3389/fvets.2025.1532422 (PMC12075304; doi:10.3389/fvets.2025.1532422)
Supplement: Supplementary file 1 [file Table_1.DOCX]

***Supplementary Material***

# Supplementary Tables

**Supplementary Table 1.** Kaplan-Meier survival analysis (Log-Rank Test) for the demographic characteristics of the study populations that survived for more than one year (365 days).

|  |  |  | **Overall survival times (days)** | |  | |
| --- | --- | --- | --- | --- | --- | --- |
|  | **Variables** | **Cases** | **Mean** | **Median** | | **p-value** |
| Canine | Sex |  |  |  | 0.7 | |
|  | Male | 727 | 4060 | 4390 |  | |
|  | Female | 967 | 4078 | 4395 |  | |
|  | Neuter Status  (in Males) |  |  |  | 0.07 | |
|  | Not Neutered | 166 | 3734 | 4180 |  | |
|  | Neutered | 561 | 4157 | 4475 |  | |
|  | Spay Status  (in Females) |  |  |  | < 0.001 | |
|  | Not Spayed | 523 | 3492 | 3659 |  | |
|  | Spayed | 444 | 4770 | 5083 |  | |
| Feline | Sex |  |  |  | 0.2 | |
|  | Male | 446 | 2687 | 2349 |  | |
|  | Female | 391 | 2891 | 2845 |  | |
|  | Neuter Status  (in Males) |  |  |  | 0.5 | |
|  | Not Neutered | 53 | 2190 | 1461 |  | |
|  | Neutered | 393 | 2753 | 2544 |  | |
|  | Spay Status  (in Females) |  |  |  | < 0.001 | |
|  | Not Spayed | 76 | 1907 | 1092 |  | |
|  | Spayed | 315 | 3128 | 2985 |  | |

**Supplementary Table 2.** Parameters used in the life tables.

| Parameter | Definition | Equation |
| --- | --- | --- |
| $d_{x}$ | Numbers of dogs or cats dying in the year interval between x and x+1 |  |
| $l_{x}$ | Numbers of dogs or cats living at year x | $\Sigma_{i=x}^{n}d_{i}$ |
| $q^{x}$ | Probability of dogs or cats dying in the year interval between x and x+1 | $q^{x}$ = $d_{x}$／$l_{x}$ |
| $a^{x}$ | Mean fraction of last year of life lived by dogs or cats died in the year interval x and x+1 | $a^{x}$= $\frac{\Sigma(lifespan*-x)}{d_{x}}$ |
| $L_{x}$ | Number of dog-years or cat-years lived in the year interval between x and x+1 | $L_{x}$ = ($l_{x}$─ $d_{x}$) + $a^{x}$ $\times$ $d_{x}$ |
| $T_{x}$ | Number of dog-years or cat-years lived beyond year x | $\Sigma_{i=x}^{n}L_{i}$ |
| $e^{x}$ | Life expectancy at year x | $e^{x}$= $T_{x}$／$l_{x}$ |

* lifespan of dogs dying in the year interval between x and x+1

**Supplementary Table 3.** Life table of the study population of dogs.

| **age(year)** | **Number of dogs**  **died in** | **Number of dogs**  **living at x** | **Probability of dogs dying in** | **Mean fraction of last year of life lived by dogs died in** | **Number of dog- years lived in** | **Number of dog- years lived beyond x** | **Life expectancy at x** |
| --- | --- | --- | --- | --- | --- | --- | --- |
|  | $d_{x}$ | $l_{x}$ | $q^{x}$ | $a^{x}$ | $L_{x}$ | $T_{x}$ | $e^{x}$ |
| 0-1 | 325 | 2024 | 0.161 | 0.334 | 1807.55 | 19005.816 | 9.39 |
| 1-2 | 90 | 1699 | 0.053 | 0.394 | 1644.46 | 17198.266 | 10.123 |
| 2-3 | 52 | 1609 | 0.032 | 0.372 | 1576.344 | 15553.806 | 9.667 |
| 3-4 | 71 | 1557 | 0.046 | 0.357 | 1511.347 | 13977.462 | 8.977 |
| 4-5 | 50 | 1486 | 0.034 | 0.355 | 1453.75 | 12466.115 | 8.389 |
| 5-6 | 52 | 1436 | 0.036 | 0.454 | 1407.608 | 11012.365 | 7.669 |
| 6-7 | 55 | 1384 | 0.04 | 0.404 | 1351.22 | 9604.757 | 6.94 |
| 7-8 | 72 | 1329 | 0.054 | 0.364 | 1283.208 | 8253.537 | 6.21 |
| 8-9 | 73 | 1257 | 0.058 | 0.413 | 1214.149 | 6970.329 | 5.545 |
| 9-10 | 85 | 1184 | 0.072 | 0.421 | 1134.785 | 5756.18 | 4.862 |
| 10-11 | 120 | 1099 | 0.109 | 0.369 | 1023.28 | 4621.395 | 4.205 |
| 11-12 | 110 | 979 | 0.112 | 0.418 | 914.98 | 3598.115 | 3.675 |
| 12-13 | 119 | 869 | 0.137 | 0.428 | 800.932 | 2683.135 | 3.088 |
| 13-14 | 150 | 750 | 0.2 | 0.365 | 654.75 | 1882.203 | 2.51 |
| 14-15 | 151 | 600 | 0.252 | 0.368 | 504.568 | 1227.453 | 2.046 |
| 15-16 | 174 | 449 | 0.388 | 0.359 | 337.466 | 722.885 | 1.61 |
| 16-17 | 113 | 275 | 0.411 | 0.376 | 204.488 | 385.419 | 1.402 |
| 17-18 | 86 | 162 | 0.531 | 0.331 | 104.466 | 180.931 | 1.117 |
| 18-19 | 45 | 76 | 0.592 | 0.3 | 44.5 | 76.465 | 1.006 |
| 19-20 | 16 | 31 | 0.516 | 0.384 | 21.144 | 31.965 | 1.031 |
| 20-21 | 9 | 15 | 0.6 | 0.197 | 7.773 | 10.821 | 0.721 |
| 21-22 | 4 | 6 | 0.667 | 0.201 | 2.804 | 3.048 | 0.508 |
| 22-23 | 2 | 2 | 1 | 0.122 | 0.244 | 0.244 | 0.122 |

**Supplementary Table 4.** Life table of the study population of cats.

| **age(year)** | **Number of cats**  **died in** | **Number of cats**  **living at x** | **Probability of cats dying in** | **Mean fraction of last year of life lived by cats died in** | **Number of cat- years lived in** | **Number of cat- years lived beyond x** | **Life expectancy at x** |
| --- | --- | --- | --- | --- | --- | --- | --- |
|  | $d_{x}$ | $l_{x}$ | $q^{x}$ | $a^{x}$ | $L_{x}$ | $T_{x}$ | $e^{x}$ |
| 0-1 | 366 | 1213 | 0.302 | 0.386 | 988.276 | 6530.743 | 5.384 |
| 1-2 | 116 | 847 | 0.137 | 0.322 | 768.352 | 5542.467 | 6.544 |
| 2-3 | 72 | 731 | 0.098 | 0.29 | 679.88 | 4774.115 | 6.531 |
| 3-4 | 52 | 659 | 0.079 | 0.378 | 626.656 | 4094.235 | 6.213 |
| 4-5 | 57 | 607 | 0.094 | 0.278 | 565.846 | 3467.579 | 5.713 |
| 5-6 | 65 | 550 | 0.118 | 0.36 | 508.4 | 2901.733 | 5.276 |
| 6-7 | 52 | 485 | 0.107 | 0.323 | 449.796 | 2393.333 | 4.935 |
| 7-8 | 53 | 433 | 0.122 | 0.344 | 398.232 | 1943.537 | 4.489 |
| 8-9 | 46 | 380 | 0.121 | 0.284 | 347.064 | 1545.305 | 4.067 |
| 9-10 | 49 | 334 | 0.147 | 0.359 | 302.591 | 1198.241 | 3.588 |
| 10-11 | 61 | 285 | 0.214 | 0.278 | 240.958 | 895.65 | 3.143 |
| 11-12 | 49 | 224 | 0.219 | 0.33 | 191.17 | 654.692 | 2.923 |
| 12-13 | 35 | 175 | 0.2 | 0.274 | 149.59 | 463.522 | 2.649 |
| 13-14 | 38 | 140 | 0.271 | 0.319 | 114.122 | 313.932 | 2.242 |
| 14-15 | 31 | 102 | 0.304 | 0.34 | 81.54 | 199.81 | 1.959 |
| 15-16 | 25 | 71 | 0.352 | 0.261 | 52.525 | 118.27 | 1.666 |
| 16-17 | 21 | 46 | 0.457 | 0.405 | 33.505 | 65.745 | 1.429 |
| 17-18 | 14 | 25 | 0.56 | 0.463 | 17.482 | 32.24 | 1.29 |
| 18-19 | 6 | 11 | 0.545 | 0.471 | 7.826 | 14.758 | 1.342 |
| 19-20 | 2 | 5 | 0.4 | 0.366 | 3.732 | 6.932 | 1.386 |
| 20-21 | 2 | 3 | 0.667 | 0.696 | 2.392 | 3.2 | 1.067 |
| 21-22 | 1 | 1 | 1 | 0.808 | 0.808 | 0.808 | 0.808 |

**Supplementary Table 5.** Life table of intact dogs.

| **age(year)** | **Number of dogs**  **died in** | **Number of dogs**  **living at x** | **Probability of dogs dying in** | **Mean fraction of last year of life lived by dogs died in** | **Number of dog- years lived in** | **Number of dog- years lived beyond x** | **Life expectancy at x** |
| --- | --- | --- | --- | --- | --- | --- | --- |
|  | $d_{x}$ | $l_{x}$ | $q^{x}$ | $a^{x}$ | $L_{x}$ | $T_{x}$ | $e^{x}$ |
| 0-1 | 285 | 979 | 0.291 | 0.32 | 785.2 | 6798.082 | 6.944 |
| 1-2 | 65 | 694 | 0.094 | 0.404 | 655.26 | 6012.882 | 8.664 |
| 2-3 | 31 | 629 | 0.049 | 0.341 | 608.571 | 5357.622 | 8.518 |
| 3-4 | 48 | 598 | 0.08 | 0.359 | 567.232 | 4749.051 | 7.942 |
| 4-5 | 31 | 550 | 0.056 | 0.398 | 531.338 | 4181.819 | 7.603 |
| 5-6 | 26 | 519 | 0.05 | 0.51 | 506.26 | 3650.481 | 7.034 |
| 6-7 | 26 | 493 | 0.053 | 0.449 | 478.674 | 3144.221 | 6.378 |
| 7-8 | 30 | 467 | 0.064 | 0.342 | 447.26 | 2665.547 | 5.708 |
| 8-9 | 29 | 437 | 0.066 | 0.415 | 420.035 | 2218.287 | 5.076 |
| 9-10 | 38 | 408 | 0.093 | 0.337 | 382.806 | 1798.252 | 4.407 |
| 10-11 | 54 | 370 | 0.146 | 0.383 | 336.682 | 1415.446 | 3.826 |
| 11-12 | 36 | 316 | 0.114 | 0.416 | 294.976 | 1078.764 | 3.414 |
| 12-13 | 45 | 280 | 0.161 | 0.404 | 253.18 | 783.788 | 2.799 |
| 13-14 | 55 | 235 | 0.234 | 0.309 | 196.995 | 530.608 | 2.258 |
| 14-15 | 46 | 180 | 0.256 | 0.328 | 149.088 | 333.613 | 1.853 |
| 15-16 | 61 | 134 | 0.455 | 0.237 | 87.457 | 184.525 | 1.377 |
| 16-17 | 29 | 73 | 0.397 | 0.313 | 53.077 | 97.068 | 1.33 |
| 17-18 | 24 | 44 | 0.545 | 0.201 | 24.824 | 43.991 | 1 |
| 18-19 | 11 | 20 | 0.55 | 0.205 | 11.255 | 19.167 | 0.958 |
| 19-20 | 4 | 9 | 0.444 | 0.37 | 6.48 | 7.912 | 0.879 |
| 20-21 | 4 | 5 | 0.8 | 0.108 | 1.432 | 1.432 | 0.286 |
| 21-22 | 1 | 1 | 1 | 0 | 0 | 0 | 0 |

**Supplementary Table 6.** Life table of spay or neutered dogs.

| **age(year)** | **Number of dogs**  **died in** | **Number of dogs**  **living at x** | **Probability of dogs dying in** | **Mean fraction of last year of life lived by dogs died in** | **Number of dog- years lived in** | **Number of dog- years lived beyond x** | **Life expectancy at x** |
| --- | --- | --- | --- | --- | --- | --- | --- |
|  | $d_{x}$ | $l_{x}$ | $q^{x}$ | $a^{x}$ | $L_{x}$ | $T_{x}$ | $e^{x}$ |
| 0-1 | 40 | 1045 | 0.038 | 0.435 | 1022.4 | 12207.997 | 11.682 |
| 1-2 | 25 | 1005 | 0.025 | 0.369 | 989.225 | 11185.597 | 11.13 |
| 2-3 | 21 | 980 | 0.021 | 0.418 | 967.778 | 10196.372 | 10.404 |
| 3-4 | 23 | 959 | 0.024 | 0.353 | 944.119 | 9228.594 | 9.623 |
| 4-5 | 19 | 936 | 0.02 | 0.284 | 922.396 | 8284.475 | 8.851 |
| 5-6 | 26 | 917 | 0.028 | 0.398 | 901.348 | 7362.079 | 8.028 |
| 6-7 | 29 | 891 | 0.033 | 0.363 | 872.527 | 6460.731 | 7.251 |
| 7-8 | 42 | 862 | 0.049 | 0.38 | 835.96 | 5588.204 | 6.483 |
| 8-9 | 44 | 820 | 0.054 | 0.412 | 794.128 | 4752.244 | 5.795 |
| 9-10 | 47 | 776 | 0.061 | 0.489 | 751.983 | 3958.116 | 5.101 |
| 10-11 | 66 | 729 | 0.091 | 0.358 | 686.628 | 3206.133 | 4.398 |
| 11-12 | 74 | 663 | 0.112 | 0.419 | 620.006 | 2519.505 | 3.8 |
| 12-13 | 74 | 589 | 0.126 | 0.442 | 547.708 | 1899.499 | 3.225 |
| 13-14 | 95 | 515 | 0.184 | 0.398 | 457.81 | 1351.791 | 2.625 |
| 14-15 | 105 | 420 | 0.25 | 0.386 | 355.53 | 893.981 | 2.129 |
| 15-16 | 113 | 315 | 0.359 | 0.425 | 250.025 | 538.451 | 1.709 |
| 16-17 | 84 | 202 | 0.416 | 0.398 | 151.432 | 288.426 | 1.428 |
| 17-18 | 62 | 118 | 0.525 | 0.382 | 79.684 | 136.994 | 1.161 |
| 18-19 | 34 | 56 | 0.607 | 0.331 | 33.254 | 57.31 | 1.023 |
| 19-20 | 12 | 22 | 0.545 | 0.389 | 14.668 | 24.056 | 1.093 |
| 20-21 | 5 | 10 | 0.5 | 0.268 | 6.34 | 9.388 | 0.939 |
| 21-22 | 3 | 5 | 0.6 | 0.268 | 2.804 | 3.048 | 0.61 |
| 22-23 | 2 | 2 | 1 | 0.122 | 0.244 | 0.244 | 0.122 |

**Supplementary Table 7.** Life table of male dogs.

| **age(year)** | **Number of dogs**  **died in** | **Number of dogs**  **living at x** | **Probability of dogs dying in** | **Mean fraction of last year of life lived by dogs died in** | **Number of dog- years lived in** | **Number of dog- years lived beyond x** | **Life expectancy at x** |
| --- | --- | --- | --- | --- | --- | --- | --- |
|  | $d_{x}$ | $l_{x}$ | $q^{x}$ | $a^{x}$ | $L_{x}$ | $T_{x}$ | $e^{x}$ |
| 0-1 | 167 | 895 | 0.187 | 0.328 | 782.776 | 8142.74 | 9.098 |
| 1-2 | 35 | 728 | 0.048 | 0.369 | 705.915 | 7359.964 | 10.11 |
| 2-3 | 23 | 693 | 0.033 | 0.364 | 678.372 | 6654.049 | 9.602 |
| 3-4 | 26 | 670 | 0.039 | 0.353 | 653.178 | 5975.677 | 8.919 |
| 4-5 | 24 | 644 | 0.037 | 0.36 | 628.64 | 5322.499 | 8.265 |
| 5-6 | 26 | 620 | 0.042 | 0.368 | 603.568 | 4693.859 | 7.571 |
| 6-7 | 20 | 594 | 0.034 | 0.253 | 579.06 | 4090.291 | 6.886 |
| 7-8 | 38 | 574 | 0.066 | 0.434 | 552.492 | 3511.231 | 6.117 |
| 8-9 | 32 | 536 | 0.06 | 0.472 | 519.104 | 2958.739 | 5.52 |
| 9-10 | 32 | 504 | 0.063 | 0.423 | 485.536 | 2439.635 | 4.841 |
| 10-11 | 55 | 472 | 0.117 | 0.385 | 438.175 | 1954.099 | 4.14 |
| 11-12 | 43 | 417 | 0.103 | 0.383 | 390.469 | 1515.924 | 3.635 |
| 12-13 | 56 | 374 | 0.15 | 0.442 | 342.752 | 1125.455 | 3.009 |
| 13-14 | 72 | 318 | 0.226 | 0.37 | 272.64 | 782.703 | 2.461 |
| 14-15 | 63 | 246 | 0.256 | 0.344 | 204.672 | 510.063 | 2.073 |
| 15-16 | 70 | 183 | 0.383 | 0.397 | 140.79 | 305.391 | 1.669 |
| 16-17 | 46 | 113 | 0.407 | 0.401 | 85.446 | 164.601 | 1.457 |
| 17-18 | 33 | 67 | 0.493 | 0.332 | 44.956 | 79.155 | 1.181 |
| 18-19 | 19 | 34 | 0.559 | 0.299 | 20.681 | 34.199 | 1.006 |
| 19-20 | 10 | 15 | 0.667 | 0.315 | 8.15 | 13.518 | 0.901 |
| 20-21 | 2 | 5 | 0.4 | 0.26 | 3.52 | 5.368 | 1.074 |
| 21-22 | 2 | 3 | 0.667 | 0.353 | 1.706 | 1.848 | 0.616 |
| 22-23 | 1 | 1 | 1 | 0.142 | 0.142 | 0.142 | 0.142 |

**Supplementary Table 8.** Life table of female dogs.

| **age(year)** | **Number of dogs**  **died in** | **Number of dogs**  **living at x** | **Probability of dogs dying in** | **Mean fraction of last year of life lived by dogs died in** | **Number of dog- years lived in** | **Number of dog- years lived beyond x** | **Life expectancy at x** |
| --- | --- | --- | --- | --- | --- | --- | --- |
|  | $d_{x}$ | $l_{x}$ | $q^{x}$ | $a^{x}$ | $L_{x}$ | $T_{x}$ | $e^{x}$ |
| 0-1 | 158 | 1129 | 0.14 | 0.341 | 1024.878 | 10863.465 | 9.622 |
| 1-2 | 55 | 971 | 0.057 | 0.41 | 938.55 | 9838.587 | 10.132 |
| 2-3 | 29 | 916 | 0.032 | 0.378 | 897.962 | 8900.037 | 9.716 |
| 3-4 | 45 | 887 | 0.051 | 0.36 | 858.2 | 8002.075 | 9.022 |
| 4-5 | 26 | 842 | 0.031 | 0.35 | 825.1 | 7143.875 | 8.484 |
| 5-6 | 26 | 816 | 0.032 | 0.54 | 804.04 | 6318.775 | 7.744 |
| 6-7 | 35 | 790 | 0.044 | 0.49 | 772.15 | 5514.735 | 6.981 |
| 7-8 | 34 | 755 | 0.045 | 0.286 | 730.724 | 4742.585 | 6.282 |
| 8-9 | 41 | 721 | 0.057 | 0.367 | 695.047 | 4011.861 | 5.564 |
| 9-10 | 53 | 680 | 0.078 | 0.42 | 649.26 | 3316.814 | 4.878 |
| 10-11 | 65 | 627 | 0.104 | 0.357 | 585.205 | 2667.554 | 4.254 |
| 11-12 | 67 | 562 | 0.119 | 0.44 | 524.48 | 2082.349 | 3.705 |
| 12-13 | 63 | 495 | 0.127 | 0.415 | 458.145 | 1557.869 | 3.147 |
| 13-14 | 78 | 432 | 0.181 | 0.361 | 382.158 | 1099.724 | 2.546 |
| 14-15 | 88 | 354 | 0.249 | 0.386 | 299.968 | 717.566 | 2.027 |
| 15-16 | 104 | 266 | 0.391 | 0.334 | 196.736 | 417.598 | 1.57 |
| 16-17 | 67 | 162 | 0.414 | 0.359 | 119.053 | 220.862 | 1.363 |
| 17-18 | 53 | 95 | 0.558 | 0.331 | 59.543 | 101.809 | 1.072 |
| 18-19 | 26 | 42 | 0.619 | 0.301 | 23.826 | 42.266 | 1.006 |
| 19-20 | 6 | 16 | 0.375 | 0.498 | 12.988 | 18.44 | 1.153 |
| 20-21 | 7 | 10 | 0.7 | 0.179 | 4.253 | 5.452 | 0.545 |
| 21-22 | 2 | 3 | 0.667 | 0.049 | 1.098 | 1.199 | 0.4 |
| 22-23 | 1 | 1 | 1 | 0.101 | 0.101 | 0.101 | 0.101 |

**Supplementary Table 9.** Life table of pure-breed dogs.

| **age(year)** | **Number of dogs**  **died in** | **Number of dogs**  **living at x** | **Probability of dogs dying in** | **Mean fraction of last year of life lived by dogs died in** | **Number of dog- years lived in** | **Number of dog- years lived beyond x** | **Life expectancy at x** |
| --- | --- | --- | --- | --- | --- | --- | --- |
|  | $d_{x}$ | $l_{x}$ | $q^{x}$ | $a^{x}$ | $L_{x}$ | $T_{x}$ | $e^{x}$ |
| 0-1 | 305 | 1823 | 0.167 | 0.338 | 1621.09 | 16737.365 | 9.181 |
| 1-2 | 85 | 1518 | 0.056 | 0.39 | 1466.15 | 15116.275 | 9.958 |
| 2-3 | 47 | 1433 | 0.033 | 0.376 | 1403.672 | 13650.125 | 9.526 |
| 3-4 | 68 | 1386 | 0.049 | 0.36 | 1342.48 | 12246.453 | 8.836 |
| 4-5 | 46 | 1318 | 0.035 | 0.354 | 1288.284 | 10903.973 | 8.273 |
| 5-6 | 48 | 1272 | 0.038 | 0.456 | 1245.888 | 9615.689 | 7.56 |
| 6-7 | 50 | 1224 | 0.041 | 0.401 | 1194.05 | 8369.801 | 6.838 |
| 7-8 | 67 | 1174 | 0.057 | 0.365 | 1131.455 | 7175.751 | 6.112 |
| 8-9 | 68 | 1107 | 0.061 | 0.431 | 1068.308 | 6044.296 | 5.46 |
| 9-10 | 80 | 1039 | 0.077 | 0.405 | 991.4 | 4975.988 | 4.789 |
| 10-11 | 110 | 959 | 0.115 | 0.365 | 889.15 | 3984.588 | 4.155 |
| 11-12 | 98 | 849 | 0.115 | 0.412 | 791.376 | 3095.438 | 3.646 |
| 12-13 | 105 | 751 | 0.14 | 0.418 | 689.89 | 2304.062 | 3.068 |
| 13-14 | 124 | 646 | 0.192 | 0.351 | 565.524 | 1614.172 | 2.499 |
| 14-15 | 138 | 522 | 0.264 | 0.374 | 435.612 | 1048.648 | 2.009 |
| 15-16 | 147 | 384 | 0.383 | 0.37 | 291.39 | 613.036 | 1.596 |
| 16-17 | 97 | 237 | 0.409 | 0.392 | 178.024 | 321.646 | 1.357 |
| 17-18 | 77 | 140 | 0.55 | 0.325 | 88.025 | 143.622 | 1.026 |
| 18-19 | 42 | 63 | 0.667 | 0.296 | 33.432 | 55.597 | 0.882 |
| 19-20 | 10 | 21 | 0.476 | 0.366 | 14.66 | 22.165 | 1.055 |
| 20-21 | 6 | 11 | 0.545 | 0.1 | 5.6 | 7.505 | 0.682 |
| 21-22 | 4 | 5 | 0.8 | 0.201 | 1.804 | 1.905 | 0.381 |
| 22-23 | 1 | 1 | 1 | 0.101 | 0.101 | 0.101 | 0.101 |

**Supplementary Table 10.** Life table of mix-breed dogs.

| **age(year)** | **Number of dogs**  **died in** | **Number of dogs**  **living at x** | **Probability of dogs dying in** | **Mean fraction of last year of life lived by dogs died in** | **Number of dog- years lived in** | **Number of dog- years lived beyond x** | **Life expectancy at x** |
| --- | --- | --- | --- | --- | --- | --- | --- |
|  | $d_{x}$ | $l_{x}$ | $q^{x}$ | $a^{x}$ | $L_{x}$ | $T_{x}$ | $e^{x}$ |
| 0-1 | 20 | 201 | 0.1 | 0.276 | 186.52 | 2267.584 | 11.282 |
| 1-2 | 5 | 181 | 0.028 | 0.461 | 178.305 | 2081.064 | 11.498 |
| 2-3 | 5 | 176 | 0.028 | 0.33 | 172.65 | 1902.759 | 10.811 |
| 3-4 | 3 | 171 | 0.018 | 0.283 | 168.849 | 1730.109 | 10.118 |
| 4-5 | 4 | 168 | 0.024 | 0.361 | 165.444 | 1561.26 | 9.293 |
| 5-6 | 4 | 164 | 0.024 | 0.436 | 161.744 | 1395.816 | 8.511 |
| 6-7 | 5 | 160 | 0.031 | 0.435 | 157.175 | 1234.072 | 7.713 |
| 7-8 | 5 | 155 | 0.032 | 0.352 | 151.76 | 1076.897 | 6.948 |
| 8-9 | 5 | 150 | 0.033 | 0.164 | 145.82 | 925.137 | 6.168 |
| 9-10 | 5 | 145 | 0.034 | 0.683 | 143.415 | 779.317 | 5.375 |
| 10-11 | 10 | 140 | 0.071 | 0.413 | 134.13 | 635.902 | 4.542 |
| 11-12 | 12 | 130 | 0.092 | 0.465 | 123.58 | 501.772 | 3.86 |
| 12-13 | 14 | 118 | 0.119 | 0.504 | 111.056 | 378.192 | 3.205 |
| 13-14 | 26 | 104 | 0.25 | 0.432 | 89.232 | 267.136 | 2.569 |
| 14-15 | 13 | 78 | 0.167 | 0.304 | 68.952 | 177.904 | 2.281 |
| 15-16 | 27 | 65 | 0.415 | 0.301 | 46.127 | 108.952 | 1.676 |
| 16-17 | 16 | 38 | 0.421 | 0.278 | 26.448 | 62.825 | 1.653 |
| 17-18 | 9 | 22 | 0.409 | 0.39 | 16.51 | 36.377 | 1.654 |
| 18-19 | 3 | 13 | 0.231 | 0.359 | 11.077 | 19.867 | 1.528 |
| 19-20 | 6 | 10 | 0.6 | 0.413 | 6.478 | 8.79 | 0.879 |
| 20-21 | 3 | 4 | 0.75 | 0.39 | 2.17 | 2.312 | 0.578 |
| 21-22 | 0 | 1 | 0 | 0 | 1.000 | 1.142 | 1.142 |
| 22-23 | 1 | 1 | 1 | 0.142 | 0.142 | 0.142 | 0.142 |

**Supplementary Table 11.** Life table of intact cats.

| **age(year)** | **Number of cats**  **died in** | **Number of cats**  **living at x** | **Probability of cats dying in** | **Mean fraction of last year of life lived by cats died in** | **Number of cat- years lived in** | **Number of cat- years lived beyond x** | **Life expectancy at x** |
| --- | --- | --- | --- | --- | --- | --- | --- |
|  | $d_{x}$ | $l_{x}$ | $q^{x}$ | $a^{x}$ | $L_{x}$ | $T_{x}$ | $e^{x}$ |
| 0-1 | 312 | 444 | 0.703 | 0.334 | 236.208 | 822.475 | 1.852 |
| 1-2 | 45 | 132 | 0.341 | 0.22 | 96.9 | 586.267 | 4.441 |
| 2-3 | 18 | 87 | 0.207 | 0.276 | 73.968 | 489.367 | 5.625 |
| 3-4 | 9 | 69 | 0.13 | 0.206 | 61.854 | 415.399 | 6.02 |
| 4-5 | 6 | 60 | 0.1 | 0.15 | 54.9 | 353.545 | 5.892 |
| 5-6 | 8 | 54 | 0.148 | 0.25 | 48 | 298.645 | 5.53 |
| 6-7 | 6 | 46 | 0.13 | 0.433 | 42.598 | 250.645 | 5.449 |
| 7-8 | 2 | 40 | 0.05 | 0.792 | 39.584 | 208.047 | 5.201 |
| 8-9 | 3 | 38 | 0.079 | 0.232 | 35.696 | 168.463 | 4.433 |
| 9-10 | 7 | 35 | 0.2 | 0.311 | 30.177 | 132.767 | 3.793 |
| 10-11 | 4 | 28 | 0.143 | 0.405 | 25.62 | 102.59 | 3.664 |
| 11-12 | 7 | 24 | 0.292 | 0.432 | 20.024 | 76.97 | 3.207 |
| 12-13 | 3 | 17 | 0.176 | 0.12 | 14.36 | 56.946 | 3.35 |
| 13-14 | 3 | 14 | 0.214 | 0.226 | 11.678 | 42.586 | 3.042 |
| 14-15 | 4 | 11 | 0.364 | 0.171 | 7.684 | 30.908 | 2.81 |
| 15-16 | 2 | 7 | 0.286 | 0.133 | 5.266 | 23.224 | 3.318 |
| 16-17 | 0 | 5 | 0 | 0 | 5 | 17.958 | 3.592 |
| 17-18 | 1 | 5 | 0.2 | 0.104 | 4.104 | 12.958 | 2.592 |
| 18-19 | 1 | 4 | 0.25 | 0.263 | 3.263 | 8.854 | 2.213 |
| 19-20 | 1 | 3 | 0.333 | 0.334 | 2.334 | 5.591 | 1.864 |
| 20-21 | 1 | 2 | 0.5 | 0.449 | 1.449 | 3.257 | 1.629 |
| 21-22 | 0 | 1 | 0 | 0 | 1 | 1.808 | 1.808 |
| 22-23 | 1 | 1 | 1 | 0.808 | 0.808 | 0.808 | 0.808 |

**Supplementary Table 12.** Life table of spay or neutered cats.

| **age(year)** | **Number of cats**  **died in** | **Number of cats**  **living at x** | **Probability of cats dying in** | **Mean fraction of last year of life lived by cats died in** | **Number of cat- years lived in** | **Number of cat- years lived beyond x** | **Life expectancy at x** |
| --- | --- | --- | --- | --- | --- | --- | --- |
|  | $d_{x}$ | $l_{x}$ | $q^{x}$ | $a^{x}$ | $L_{x}$ | $T_{x}$ | $e^{x}$ |
| 0-1 | 54 | 769 | 0.07 | 0.686 | 752.044 | 5709.333 | 7.424 |
| 1-2 | 71 | 715 | 0.099 | 0.387 | 671.477 | 4957.289 | 6.933 |
| 2-3 | 54 | 644 | 0.084 | 0.294 | 605.876 | 4285.812 | 6.655 |
| 3-4 | 43 | 590 | 0.073 | 0.414 | 564.802 | 3679.936 | 6.237 |
| 4-5 | 51 | 547 | 0.093 | 0.293 | 510.943 | 3115.134 | 5.695 |
| 5-6 | 57 | 496 | 0.115 | 0.376 | 460.432 | 2604.191 | 5.25 |
| 6-7 | 46 | 439 | 0.105 | 0.309 | 407.214 | 2143.759 | 4.883 |
| 7-8 | 51 | 393 | 0.13 | 0.327 | 358.677 | 1736.545 | 4.419 |
| 8-9 | 43 | 342 | 0.126 | 0.288 | 311.384 | 1377.868 | 4.029 |
| 9-10 | 42 | 299 | 0.14 | 0.367 | 272.414 | 1066.484 | 3.567 |
| 10-11 | 57 | 257 | 0.222 | 0.269 | 215.333 | 794.07 | 3.09 |
| 11-12 | 42 | 200 | 0.21 | 0.313 | 171.146 | 578.737 | 2.894 |
| 12-13 | 32 | 158 | 0.203 | 0.289 | 135.248 | 407.591 | 2.58 |
| 13-14 | 35 | 126 | 0.278 | 0.327 | 102.445 | 272.343 | 2.161 |
| 14-15 | 27 | 91 | 0.297 | 0.365 | 73.855 | 169.898 | 1.867 |
| 15-16 | 23 | 64 | 0.359 | 0.272 | 47.256 | 96.043 | 1.501 |
| 16-17 | 21 | 41 | 0.512 | 0.405 | 28.505 | 48.787 | 1.19 |
| 17-18 | 13 | 20 | 0.65 | 0.491 | 13.383 | 20.282 | 1.014 |
| 18-19 | 5 | 7 | 0.714 | 0.512 | 4.56 | 6.899 | 0.986 |
| 19-20 | 1 | 2 | 0.5 | 0.397 | 1.397 | 2.339 | 1.17 |
| 20-21 | 1 | 1 | 1 | 0.942 | 0.942 | 0.942 | 0.942 |

**Supplementary Table 13.** Life table of male cats.

| **age(year)** | **Number of cats**  **died in** | **Number of cats**  **living at x** | **Probability of cats dying in** | **Mean fraction of last year of life lived by cats died in** | **Number of cat- years lived in** | **Number of cat- years lived beyond x** | **Life expectancy at x** |
| --- | --- | --- | --- | --- | --- | --- | --- |
|  | $d_{x}$ | $l_{x}$ | $q^{x}$ | $a^{x}$ | $L_{x}$ | $T_{x}$ | $e^{x}$ |
| 0-1 | 214 | 665 | 0.322 | 0.403 | 537.242 | 3373.562 | 5.073 |
| 1-2 | 60 | 451 | 0.133 | 0.374 | 413.44 | 2836.32 | 6.289 |
| 2-3 | 39 | 391 | 0.1 | 0.269 | 362.491 | 2422.88 | 6.197 |
| 3-4 | 37 | 352 | 0.105 | 0.408 | 330.096 | 2060.389 | 5.853 |
| 4-5 | 38 | 315 | 0.121 | 0.281 | 287.678 | 1730.293 | 5.493 |
| 5-6 | 38 | 277 | 0.137 | 0.286 | 249.868 | 1442.615 | 5.208 |
| 6-7 | 28 | 239 | 0.117 | 0.354 | 220.912 | 1192.747 | 4.991 |
| 7-8 | 24 | 211 | 0.114 | 0.34 | 195.16 | 971.835 | 4.606 |
| 8-9 | 24 | 187 | 0.128 | 0.321 | 170.704 | 776.675 | 4.153 |
| 9-10 | 21 | 163 | 0.129 | 0.357 | 149.497 | 605.971 | 3.718 |
| 10-11 | 29 | 142 | 0.204 | 0.296 | 121.584 | 456.474 | 3.215 |
| 11-12 | 27 | 113 | 0.239 | 0.435 | 97.745 | 334.89 | 2.964 |
| 12-13 | 17 | 86 | 0.198 | 0.276 | 73.692 | 237.145 | 2.758 |
| 13-14 | 17 | 69 | 0.246 | 0.298 | 57.066 | 163.453 | 2.369 |
| 14-15 | 13 | 52 | 0.25 | 0.355 | 43.615 | 106.387 | 2.046 |
| 15-16 | 15 | 39 | 0.385 | 0.22 | 27.3 | 62.772 | 1.61 |
| 16-17 | 13 | 24 | 0.542 | 0.344 | 15.472 | 35.472 | 1.478 |
| 17-18 | 5 | 11 | 0.455 | 0.61 | 9.05 | 20 | 1.818 |
| 18-19 | 2 | 6 | 0.333 | 0.208 | 4.416 | 10.95 | 1.825 |
| 19-20 | 1 | 4 | 0.25 | 0.334 | 3.334 | 6.534 | 1.634 |
| 20-21 | 2 | 3 | 0.667 | 0.696 | 2.392 | 3.2 | 1.067 |
| 21-22 | 0 | 1 | 0 | 0.000 | 1.000 | 1.808 | 1.808 |
| 22-23 | 1 | 1 | 1 | 0.808 | 0.808 | 0.808 | 0.808 |

**Supplementary Table 14.** Life table of female cats.

| **age(year)** | **Number of cats**  **died in** | **Number of cats**  **living at x** | **Probability of cats dying in** | **Mean fraction of last year of life lived by cats died in** | **Number of cat- years lived in** | **Number of cat- years lived beyond x** | **Life expectancy at x** |
| --- | --- | --- | --- | --- | --- | --- | --- |
|  | $d_{x}$ | $l_{x}$ | $q^{x}$ | $a^{x}$ | $L_{x}$ | $T_{x}$ | $e^{x}$ |
| 0-1 | 152 | 548 | 0.277 | 0.363 | 451.176 | 3157.42 | 5.762 |
| 1-2 | 56 | 396 | 0.141 | 0.267 | 354.952 | 2706.244 | 6.834 |
| 2-3 | 33 | 340 | 0.097 | 0.314 | 317.362 | 2351.292 | 6.916 |
| 3-4 | 15 | 307 | 0.049 | 0.303 | 296.545 | 2033.93 | 6.625 |
| 4-5 | 19 | 292 | 0.065 | 0.271 | 278.149 | 1737.385 | 5.95 |
| 5-6 | 27 | 273 | 0.099 | 0.465 | 258.555 | 1459.236 | 5.345 |
| 6-7 | 24 | 246 | 0.098 | 0.288 | 228.912 | 1200.681 | 4.881 |
| 7-8 | 29 | 222 | 0.131 | 0.348 | 203.092 | 971.769 | 4.377 |
| 8-9 | 22 | 193 | 0.114 | 0.245 | 176.39 | 768.677 | 3.983 |
| 9-10 | 28 | 171 | 0.164 | 0.361 | 153.108 | 592.287 | 3.464 |
| 10-11 | 32 | 143 | 0.224 | 0.262 | 119.384 | 439.179 | 3.071 |
| 11-12 | 22 | 111 | 0.198 | 0.201 | 93.422 | 319.795 | 2.881 |
| 12-13 | 18 | 89 | 0.202 | 0.272 | 75.896 | 226.373 | 2.544 |
| 13-14 | 21 | 71 | 0.296 | 0.337 | 57.077 | 150.477 | 2.119 |
| 14-15 | 18 | 50 | 0.36 | 0.328 | 37.904 | 93.4 | 1.868 |
| 15-16 | 10 | 32 | 0.312 | 0.323 | 25.23 | 55.496 | 1.734 |
| 16-17 | 8 | 22 | 0.364 | 0.504 | 18.032 | 30.266 | 1.376 |
| 17-18 | 9 | 14 | 0.643 | 0.381 | 8.429 | 12.234 | 0.874 |
| 18-19 | 4 | 5 | 0.8 | 0.602 | 3.408 | 3.805 | 0.761 |
| 19-20 | 1 | 1 | 1 | 0.397 | 0.397 | 0.397 | 0.397 |

**Supplementary Table 15.** Life table of pure-breed cats.

| **age(year)** | **Number of cats**  **died in** | **Number of cats**  **living at x** | **Probability of cats dying in** | **Mean fraction of last year of life lived by cats died in** | **Number of cat- years lived in** | **Number of cat- years lived beyond x** | **Life expectancy at x** |
| --- | --- | --- | --- | --- | --- | --- | --- |
|  | $d_{x}$ | $l_{x}$ | $q^{x}$ | $a^{x}$ | $L_{x}$ | $T_{x}$ | $e^{x}$ |
| 0-1 | 145 | 575 | 0.252 | 0.415 | 490.175 | 3416.776 | 5.942 |
| 1-2 | 48 | 430 | 0.112 | 0.402 | 401.296 | 2926.601 | 6.806 |
| 2-3 | 41 | 382 | 0.107 | 0.338 | 354.858 | 2525.305 | 6.611 |
| 3-4 | 23 | 341 | 0.067 | 0.451 | 328.373 | 2170.447 | 6.365 |
| 4-5 | 27 | 318 | 0.085 | 0.33 | 299.91 | 1842.074 | 5.793 |
| 5-6 | 31 | 291 | 0.107 | 0.458 | 274.198 | 1542.164 | 5.3 |
| 6-7 | 31 | 260 | 0.119 | 0.367 | 240.377 | 1267.966 | 4.877 |
| 7-8 | 33 | 229 | 0.144 | 0.381 | 208.573 | 1027.589 | 4.487 |
| 8-9 | 20 | 196 | 0.102 | 0.332 | 182.64 | 819.016 | 4.179 |
| 9-10 | 29 | 176 | 0.165 | 0.361 | 157.469 | 636.376 | 3.616 |
| 10-11 | 29 | 147 | 0.197 | 0.308 | 126.932 | 478.907 | 3.258 |
| 11-12 | 25 | 118 | 0.212 | 0.286 | 100.15 | 351.975 | 2.983 |
| 12-13 | 17 | 93 | 0.183 | 0.242 | 80.114 | 251.825 | 2.708 |
| 13-14 | 19 | 76 | 0.25 | 0.227 | 61.313 | 171.711 | 2.259 |
| 14-15 | 16 | 57 | 0.281 | 0.345 | 46.52 | 110.398 | 1.937 |
| 15-16 | 13 | 41 | 0.317 | 0.177 | 30.301 | 63.878 | 1.558 |
| 16-17 | 14 | 28 | 0.5 | 0.459 | 20.426 | 33.577 | 1.199 |
| 17-18 | 10 | 14 | 0.714 | 0.462 | 8.62 | 13.151 | 0.939 |
| 18-19 | 2 | 4 | 0.5 | 0.374 | 2.748 | 4.531 | 1.133 |
| 19-20 | 1 | 2 | 0.5 | 0.334 | 1.334 | 1.783 | 0.891 |
| 20-21 | 1 | 1 | 1 | 0.449 | 0.449 | 0.449 | 0.449 |

**Supplementary Table. 16.** Life table of mix-breed cats.

| **age(year)** | **Number of cats**  **died in** | **Number of cats**  **living at x** | **Probability of cats dying in** | **Mean fraction of last year of life lived by cats died in** | **Number of cat- years lived in** | **Number of cat- years lived beyond x** | **Life expectancy at x** |
| --- | --- | --- | --- | --- | --- | --- | --- |
|  | $d_{x}$ | $l_{x}$ | $q^{x}$ | $a^{x}$ | $L_{x}$ | $T_{x}$ | $e^{x}$ |
| 0-1 | 221 | 638 | 0.346 | 0.367 | 498.107 | 3114.08 | 4.881 |
| 1-2 | 68 | 417 | 0.163 | 0.265 | 367.02 | 2615.973 | 6.273 |
| 2-3 | 31 | 349 | 0.089 | 0.226 | 325.006 | 2248.953 | 6.444 |
| 3-4 | 29 | 318 | 0.091 | 0.32 | 298.28 | 1923.947 | 6.05 |
| 4-5 | 30 | 289 | 0.104 | 0.231 | 265.93 | 1625.667 | 5.625 |
| 5-6 | 34 | 259 | 0.131 | 0.272 | 234.248 | 1359.737 | 5.25 |
| 6-7 | 21 | 225 | 0.093 | 0.259 | 209.439 | 1125.489 | 5.002 |
| 7-8 | 20 | 204 | 0.098 | 0.285 | 189.7 | 916.05 | 4.49 |
| 8-9 | 26 | 184 | 0.141 | 0.248 | 164.448 | 726.35 | 3.948 |
| 9-10 | 20 | 158 | 0.127 | 0.357 | 145.14 | 561.902 | 3.556 |
| 10-11 | 32 | 138 | 0.232 | 0.252 | 114.064 | 416.762 | 3.02 |
| 11-12 | 24 | 106 | 0.226 | 0.376 | 91.024 | 302.698 | 2.856 |
| 12-13 | 18 | 82 | 0.22 | 0.304 | 69.472 | 211.674 | 2.581 |
| 13-14 | 19 | 64 | 0.297 | 0.411 | 52.809 | 142.202 | 2.222 |
| 14-15 | 15 | 45 | 0.333 | 0.334 | 35.01 | 89.393 | 1.987 |
| 15-16 | 12 | 30 | 0.4 | 0.352 | 22.224 | 54.383 | 1.813 |
| 16-17 | 7 | 18 | 0.389 | 0.296 | 13.072 | 32.159 | 1.787 |
| 17-18 | 4 | 11 | 0.364 | 0.466 | 8.864 | 19.087 | 1.735 |
| 18-19 | 4 | 7 | 0.571 | 0.519 | 5.076 | 10.223 | 1.46 |
| 19-20 | 1 | 3 | 0.333 | 0.397 | 2.397 | 5.147 | 1.716 |
| 20-21 | 1 | 2 | 0.5 | 0.942 | 1.942 | 2.75 | 1.375 |
| 21-22 | 0 | 1 | 0 | 0.000 | 1.000 | 1.808 | 1.808 |
| 22-23 | 1 | 1 | 1 | 0.808 | 0.808 | 0.808 | 0.808 |
